# Bioinformatics analysis reveals potential biomarkers associated with the occurrence of intracranial aneurysms

**DOI:** 10.1038/s41598-022-17510-7

**Published:** 2022-08-02

**Authors:** Chao Zhao, Zhiguo Ma, Junliang Shang, Xinchun Cui, Jinxing Liu, Ronghua Shi, Shuai Wang, Aihong Wu

**Affiliations:** 1 Juzhou Road Community Health Service Center, The Affiliated Rizhao People’s Hospital of Jining Medical University, Rizhao, Shandong China; 2 Department of Neurosurgery, The Affiliated Rizhao People’s Hospital of Jining Medical University, Rizhao, Shandong China; 3 Department of Neurosurgery, The 942th Hospital of Chinese PLA, Yinchuan, Ningxia China; 4 Library, Qufu Normal University, Rizhao, Shandong China; 5 School of Information Science and Engineering, Qufu Normal University, Rizhao, Shandong China

**Keywords:** Computational biology and bioinformatics, Molecular biology, Neuroscience

## Abstract

To better understand the molecular mechanisms of intracranial aneurysm (IA) pathogenesis, we used gene coexpression networks to identify hub genes and functional pathways associated with IA onset. Two Gene Expression Omnibus (GEO) datasets encompassing intracranial aneurysm tissue samples and cerebral artery control samples were included. To discover functional pathways and potential biomarkers, weighted gene coexpression network analysis was employed. Next, single-gene gene set enrichment analysis was employed to investigate the putative biological roles of the chosen genes. We also used receiver operating characteristic analysis to confirm the diagnostic results. Finally, we used a rat model to confirm the hub genes in the module of interest. The module of interest, which was designated the green module and included 115 hub genes, was the key module that was most strongly and negatively associated with IA formation. According to gene set variation analysis results, 15 immune-related pathways were significantly activated in the IA group, whereas 7 metabolic pathways were suppressed. In two GEO datasets, *SLC2A12* could distinguish IAs from control samples. Twenty-nine hub genes in the green module might be biomarkers for the occurrence of cerebral aneurysms. *SLC2A12* expression was significantly downregulated in both human and rat IA tissue. In the present study, we identified 115 hub genes related to the pathogenesis of IA onset and deduced their potential roles in various molecular pathways; this new information may contribute to the diagnosis and treatment of IAs. By external validation, the *SLC2A12* gene may play an important role. The molecular function of *SLC2A12* in the process of IA occurrence can be further studied in a rat model.

Despite clinical advancements, intracranial aneurysm (IA) remains a life-threatening disease. The rupture of IAs causes subarachnoid hemorrhage; only 60% of individuals survive a subarachnoid hemorrhage event, and significant disability is prevalent among survivors^1^. *S100A8, KLF2, CDKN2A*, and other molecular markers have been implicated in the formation, progression, and rupture of IAs^1^. However, the molecular mechanisms leading to these processes remain poorly understood. The invasive methods of endovascular intervention and surgical clipping are the main therapeutic methods used for IA; no safe and effective noninvasive methods for the treatments or prevention of IA have been identified and implemented in clinical practice. Our research aims to identify molecular biomarkers of IA and therapeutic targets for their treatment.

Expression microarray technology and bioinformatic analysis have been widely applied to the study of IA-related genetic dysfunctions in the past; these approaches have identified differentially expressed genes (DEGs) and signaling pathways leading to the pathogenesis and progression of IA. Compared to microarray methods, however, RNA sequencing (RNA-seq) provides several advantages, including multiple layers of resolution and transcriptome complexity, reduced background noise, and an expanded dynamic range of RNA expression^2^. However, previous studies have rarely compared IA samples with their tissues of origin, the normal cerebral arteries, using RNA-seq technology. Most of the published studies used branches of the external carotid arteries including the superficial temporal arteries (STAs)^1^ and the middle meningeal arteries (MMAs) (GSE46337), as controls. It is well known that different tissues are at least partially defined by their gene expression profiles^3^; this principle applies to the internal and external carotid arteries. Internal carotid arteries and external arteries are probably different at the gene transcription and protein levels because many cerebrovascular diseases are rarely detected in the external carotid arteries. To the best of our knowledge, no evidence has shown internal carotid arteries sharing the same gene expression profile as the external arteries. Therefore, new strategies should be taken in the study of IA pathogenesis and progression.

To better understand the molecular mechanisms of IA pathogenesis better, we investigated them via bioinformatics methods. In this study, two datasets containing IA tissue samples and cerebral artery control samples from the Gene Expression Omnibus (GEO)^4^ were included. Weighted gene coexpression network analysis (WGCNA) was used to investigate gene expression and trait data, identifying functional pathways and candidate biomarkers. We identified modules with clinical significance. After finding the module that was most strongly correlated with IA occurrence and identifying the hub genes in it, we conducted Gene Ontology (GO) and gene-set variation analysis (GSVA) to illustrate the molecular mechanisms of their pathogenesis. We referred to the FerrDb database and found that one hub gene *SLC2A12*, was related to the ferroptosis phenotype and has not previously been studied in IA research. Subsequently, single-gene gene set enrichment analysis (GSEA) was used to explore the potential biological functions of the selected gene *SLC2A1*2. Furthermore, we used the expression data of *SLC2A12* from normalized transcriptome expression data in GSE122897 and GSE157628 to validate its diagnostic utility by receiver operating characteristic (ROC) analysis. Upstream regulatory molecules of transcription factors (TFs) and microRNAs were surveyed in silico. The author discussed the roles of *SLC2A12* multiple signal pathways. Ultimately, the comprehensive gene expression profile of the *Rattus norvegicus* model validated candidate biomarkers.

This research identified possible pathogenesis-related biomarker genes and predicted their molecular mechanisms of intracranial aneurysm pathogenesis. Meanwhile, it presented an ideal target for animal model experiments (Fig. 1).

**Figure 1:**
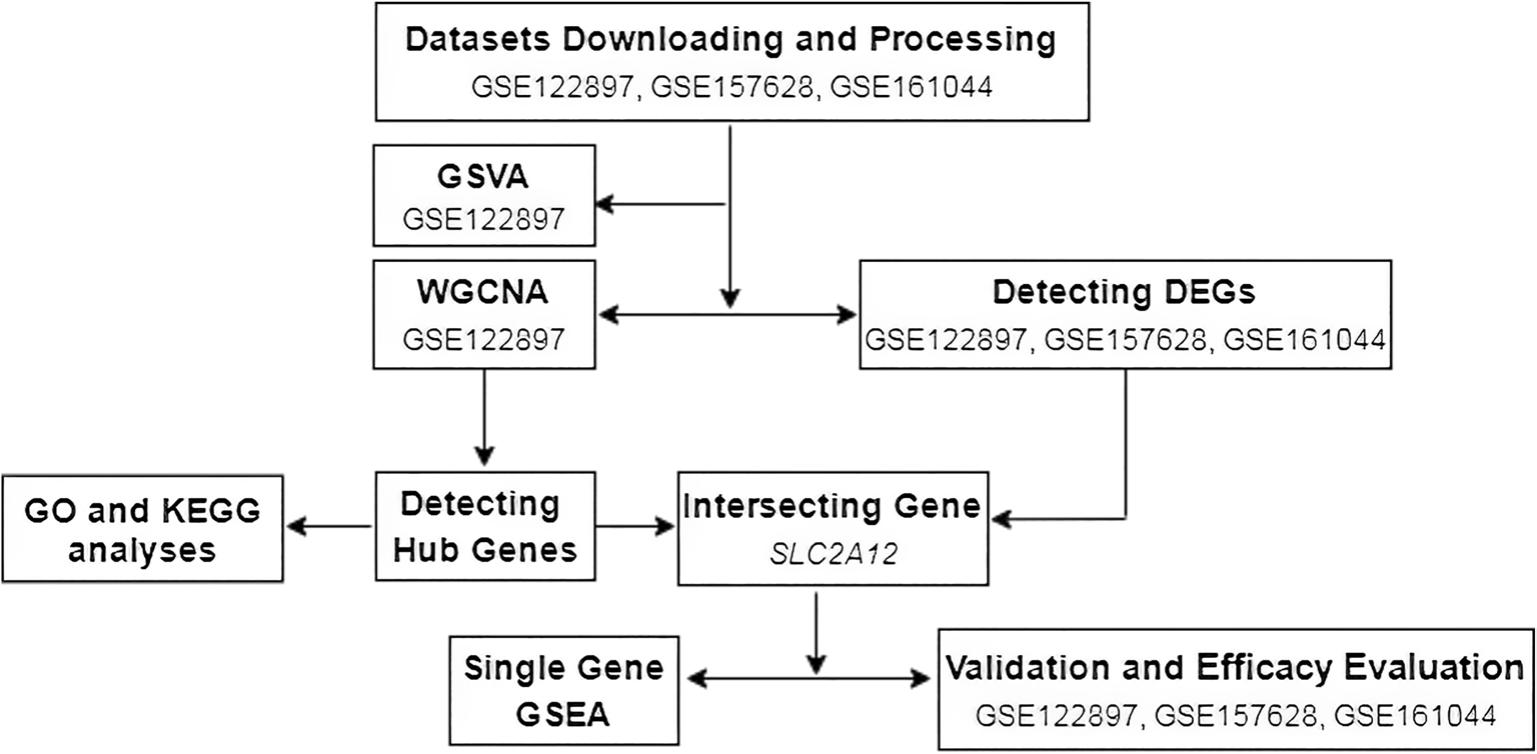
Flowchart on construction and validation of the pathogenesis-related gene of IA occurrence. IA, intracranial aneurysm; WGCNA, Weighted gene co-expression network analysis; GEO, Gene Expression Omnibus; DEGs, differential expressed genes; GO, Gene Ontology; KEGG, Kyoto Encyclopedia of Genes and Genomes; GSVA, Gene Set Variation Analysis; GSEA, Gene Set Enrichment Analysis; This flowchart was generated and edited with the free application online, diagrams.net (https://app.diagrams.net/).

## Methods

### Gene expression datasets and data processing

Two transcriptome-wide gene expression profiles of GSE122897 and GSE157628 were downloaded from GEO. The RNA-seq dataset GSE122897 contains 44 IA tissue samples and 16 control tissue samples (cerebral arteries) based on the platform of GPL16791, Illumina HiSeq 2500 (*Homo sapiens*)^5^.Two IA samples in GSE122897, IA6 (GSM3487858) and IA40 (GSM3487892), were rejected because their statuses were unknown. The raw read count profile of GSE122897 was processed by the “DESeq2” package for library normalization and independent filtering and the “AnnotationDbi” and “org.Hs.eg.db” packages for probe annotation. The total gene expression dataset, consisting of 25,807 genes was further processed for WGCNA. Clinical traits were gathered from the information of GSE122897 (Supplementary Table 1). Clinical traits included type, status, gender and age. All samples were divided into a control subgroup and IA subgroup based on whether aneurysms occurred in the type group. In addition, in the same way, ruptured or not in status group, male or female in gender group. The age of each sample, from 16 to 77, was recorded in age group.

The transcriptome-wide microarray dataset GSE157628 provided the gene expression profile of 6 IA samples and 3 controls (cerebral arteries), based on Agilent-039494 SurePrint G3 Human GE v2 8 × 60 K Microarray 039,381 (Feature Number version). The gene expression dataset of GSE157628 was processed by the limma package for background correction and normalization. DEGs in GSE157628 and GSE122897 were identified using the following criteria: P value < 0.05 and |logFC|> 1. The DEGs are listed in Supplementary output Tables 1 and 2. The GSE157628 dataset was used to validate the expression of the selected genes.

If more than one probe corresponded to one gene, the maximum expression value of each gene was used to eliminate duplicated probes. The gene expression of the above two datasets was elucidated by the genetic maximums of all corresponding probes.

### Construction of a weighted gene coexpression network

We used the whole gene transcriptome expression profile of GSE122897 to perform WGCNA. A topology network of WGCNA was constructed by the R package “WGCNA”^6^ for analysis. The adjacency matrix was transformed to a topological overlap matrix; the soft-threshold power (β) was eight when 0.85 was chosen as the correlation coefficient threshold with a scale-free R^2^ above 0.85 and a slope near 1. The minimum number of genes in the modules was 110, and the cut height threshold was 0.25 for module detection. We constructed a module-trait relationship chart and searched for the key modules that were closely related to type (IA or controls), IA status (ruptured or not), gender (male or female) and age (years).

The correlation between the eigengene module and clinical traits was used to estimate the module-trait relationship, and detect highly phenotypically related modules efficiently. We obtained the module significance (MS) by calculating the average absolute gene significance (GS) of all the genes involved in the module. GS is measured as the log10 transformation of the *P* value in the logistic regression between gene expression and clinical traits. Module member (MM) is defined as the correlation between the expression profile and each module feature value. In WGCNA, the module with the highest correlation score was defined as the key module and subjected to further analysis.

### Functional enrichment analysis

Genes in the key module of “type” indicated a significant correlation with clinical features of IA occurrence. Hub genes were defined as those with |GS|> 0.4 and |MM|> 0.8. Hub genes were selected to perform GO and Kyoto Encyclopedia of Genes and Genomes (KEGG) analyses. GO and KEGG pathway analyses were conducted to explore their biological functions utilizing the R package “clusterProfiler”^7^. GO terms and KEGG pathways were explored with adjusted *P* < 0.05 and FDR < 0.05 set as the cutoff and were visualized by the R package “GOplot”^8^.

### Identification of pathways associated with IA formation via GSVA

Using the R package “GSEABase”, we performed GSVA that was applied to the KEGG pathways enrichment described by the MSigDB which is a collection of annotated gene sets in GSEA software. The “c2.cp.kegg.v7.4.symbols.gmt”profile, containing canonical KEGG Pathways gene sets derived from the KEGG pathway database, was downloaded for this analysis. To reveal the enriched pathways within 186 gene sets, we used the GSVA package to evaluate the t score and assign pathway activity conditions between 16 control and 41 IA samples in GSE122897 with a Gaussian distribution. Moreover, the limma package was also used to find differentially expressed pathways. Adjusted *P* value < 0.05 and |logFC|> 0.3 were set as criteria.

### Validation and predictive efficacy evaluation of the key gene

Among the hub genes in one module, designated the green module, *SLC2A12* was selected as the key gene that has not been studied in IA research. We obtained the expression data of *SLC2A12* and *COL5A2* from normalized transcriptome expression data in GSE122897 and GSE157628 to construct predictive model. Then, prediction models for the ROC curve were plotted and the area under the curve (AUC) was calculated with the “pROC” package to evaluate the capability of the key gene to distinguish IA patients from controls.

### GSEA of the key gene

A “guilt by association” method has been reported to predict the functions of an unknown gene^9^. Via a guilt by association approach, we performed single-gene GSEA to explore the potential KEGG pathways of *SLC2A12* in intracranial aneurysm occurrence. The “clusterProfiler” R package was utilized for GSEA. The KEGG gene set profile "c2.cp.kegg.v7.4.symbols.gmt"was used as the reference. The correlation analysis between *SLC2A12* and all genes in GSE122897 was performed by the Spearman method, and the genes were ranked in decreasing order by the correlation score. The genelist for GSEA consisted of all the ranked genes. An adjusted P value < 0.05 was chosen as the cutoff criterion.

### Identification of the TFs, miRNAs and ferroptosis markers related to the key gene

The *SLC2A12* gene was searched in the Enrichr (http://amp.pharm.mssm.edu/Enrichr/) and hTFtarget (http://bioinfo.life.hust.edu.cn/hTFtarget/#!/) databases to obtain the interaction between *SLC2A12* and its TFs. To reduce the chance of identifying false-positives, we overlapped TFs surveyed in consensus target genes existing in ChEA, the ENCODE gene-set library and the hTFtarget database. The expression correlation between *SLC2A12* and the overlapping TF genes in GSE122897 was plotted. To identify the miRNAs targeting *SLC2A12* and those related to intracranial aneurysm occurrence, we referred to the GeneCards database (https://www.genecards.org).

The FerrDb database (http://www.zhounan.org/ferrdb/legacy/index.html/) is a manually curated resource on regulators and markers of ferroptosis as well as associations between ferroptosis and disease. All data were extracted from 784 articles in the PubMed database and annotated. We referred to FerrDb to determine the role of *SLC2A12* in the ferroptosis phenotype.

### External validation in an animal model

The gene expression matrices of the *Rattus norvegicus* model, GSE161044, were downloaded from the GEO database. It was conducted by the Illumina NextSeq 500 (*Rattus norvegicus*) System based on the platform GPL20084. This dataset consisted of 3 intracranial aneurysms (samples) and their remaining Willis circles (controls) from 3 rats by RNA-seq analysis. The raw read count profile of GSE161044 was processed by the “DESeq2” package for library normalization and independent filtering. Then, the DEGs were obtained by the criteria P value < 0.05 and |logFC|> 0.4. The DEGs are listed in Supplementary Table 2. The GSE161044 dataset was used to validate the expression of our genes of interest. Venn diagrams were then applied to identify the upregulated and downregulated common DEGs of GSE161044, GSE122897, GSE157628 and hub genes in the green module.

### Statistical analysis

The statistical significance of differences between two groups was analyzed using non-parametric test or t tests based on data distribution characteristics. All analyses were conducted using R4.1.0 software, and a *P* value < 0.05 was considered statistically significant. The R packages DESeq2, limma, WGCNA, ggplot2, export, clusterProfiler, ggstatsplot, GSEABase, GSVA, ROCR, etc., were used in this study.

## Results

### Gene coexpression networks

**Figure 2:**
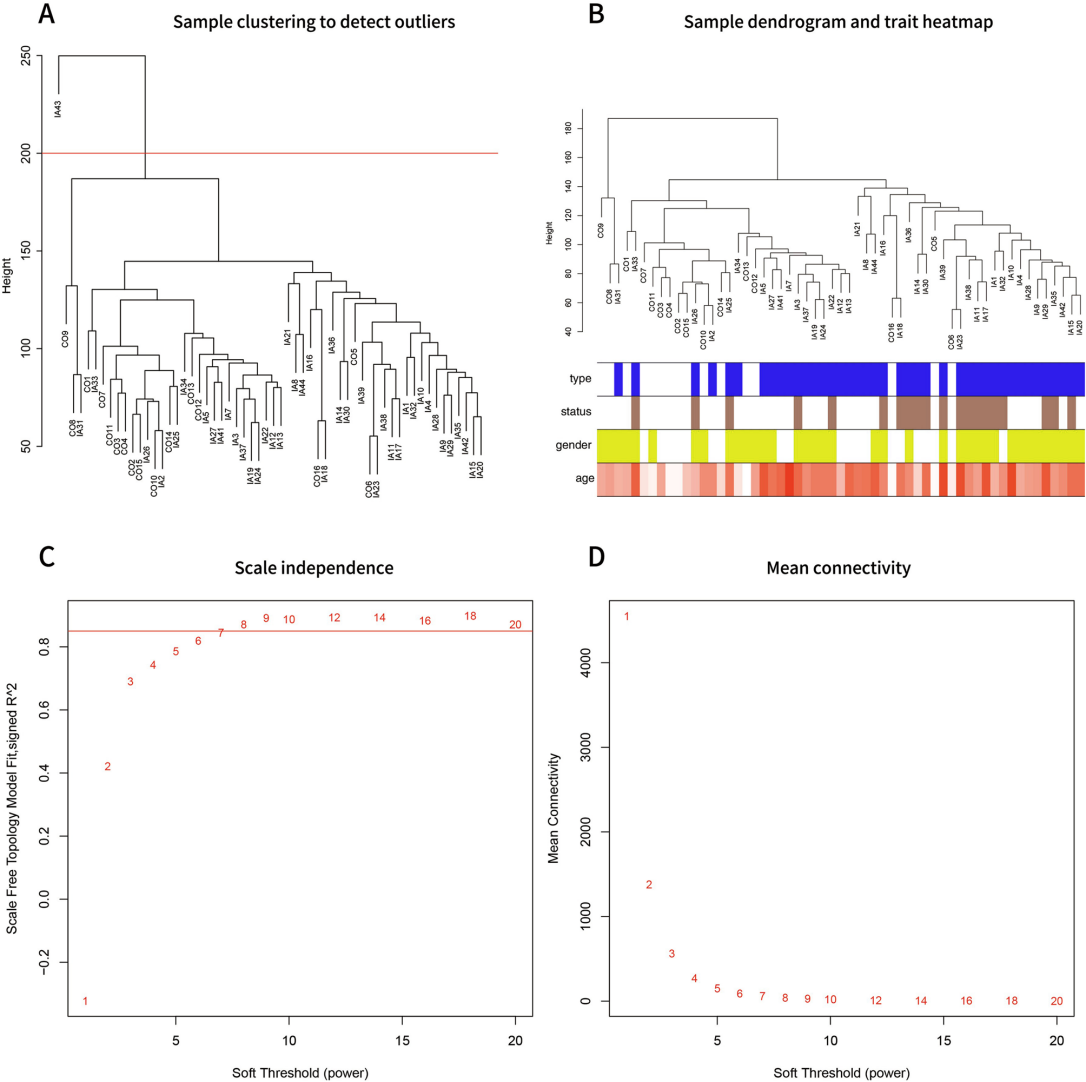
(**A**) Clustering of samples to detect outliers. (**B**) Sample dendrogram and clinical traits heatmap. blue means IA (white means control); brown means ruptured IA (white means unruptured IA);yellow means female(white means male) ; The shade of red color varies from light to dark according to the age of the patient, from 16 to 77. (**C**) Scale-free topology modelfor finding the soft-thresholding power. (**D**) Mean connectivity.

**Figure 3:**
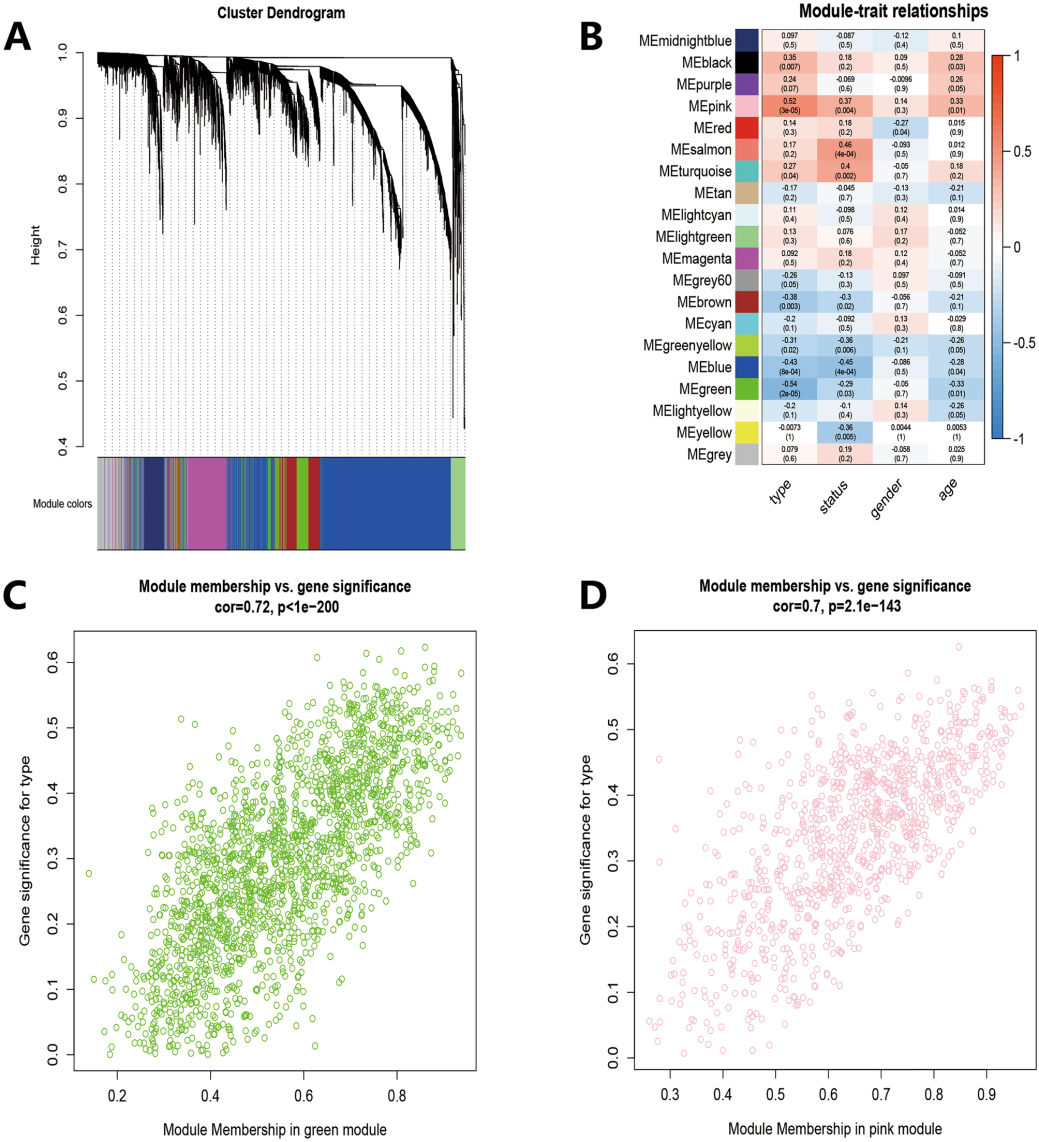
Weighted gene co-expression network analysis. (**A**) Clustering dendrogram of total genes related to IA onset. (**B**) Heatmap shows the relationships between different modules and clinical traits. (**C**) Scatter plot of module eigengenes in the green module. (**D**) Scatter plot of module eigengenes in the pink module.

After one outlier, IA43 (GSM3487895) was omitted from GSE122897, the cohort consisted of 20 ruptured IA tissues, 21 unruptured IA tissues and 16 cerebral artery controls as illustrated in Fig. 2A.The sample clustering dendrograms of the IA clinical traits (type, status, gender and age) are shown in Fig. 2B. In WGCNA, we correlated each module with clinical traits in the GSE122897 dataset by calculating the MS for each module-trait correlation. When 0.85 was used as the correlation coefficient threshold, the soft power threshold (β) was set to eight (Fig. 2C, 2D). Twenty coexpression modules were constructed by using the average linkage hierarchical clustering algorithm (Fig. 3A). Non-clustered genes were gathered in the gray module. No modules manifested strong correlations (|r|> 0.8) with clinical traits, except that the green module (r = -0.54, P = 3e−5 < 0.01) and the pink module (r = 0.52, P = 3e−5 < 0.01) showed modest correlations with type (Fig. 3B). After comparing the relationships between GS and MM, we considered the green module (correlation coefficient = 0.72, P = 2 × 10^−200^; Fig. 3C) to be the key module that was most strongly and negatively associated with IA formation and was analyzed further in detail. Meanwhile, the pink module was the most positively associated (Fig. 3D).The green module contained 1887 genes, of which 115 genes were identified as hub genes based on the cutoff criteria (|MM|> 0.8 and |GS|> 0.4) (Supplementary output Table 3).

In the pink module, 130 hub genes were identified (Supplementary output Table 4). *COL5A2* and *VIPR1* overlapped the hub genes and the differentially expressed genes of GSE157628 and GSE122897 (Supplementary Image 1).

### Functional enrichment analysis of the hub genes

**Figure 4:**
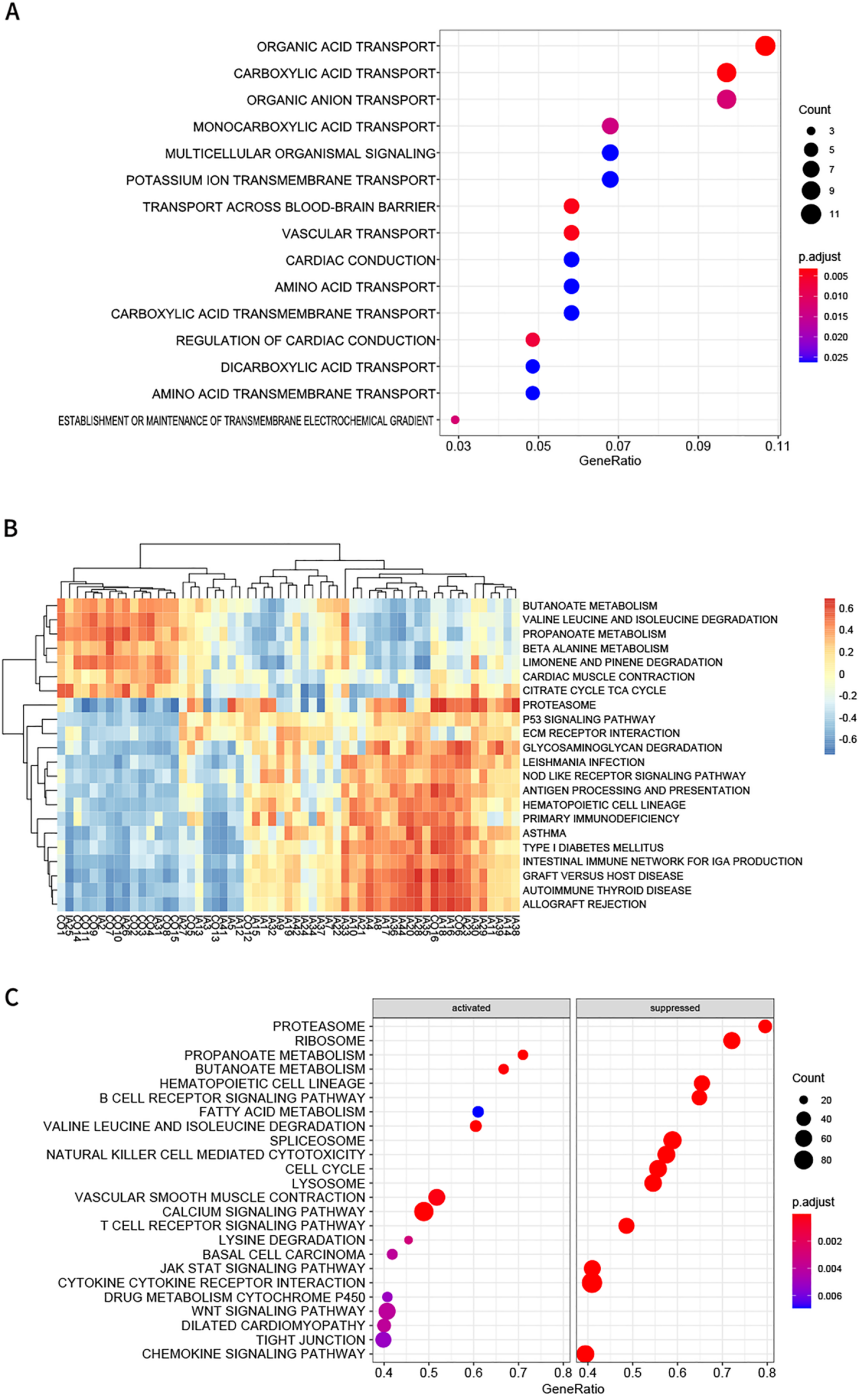
Functional enrichment of the green module. (**A**) Bubble plots of biological process of GO analysis. (**B**) Heatmap of differentially KEGG pathways via GSVA. (**C**) Heatmap of *SLC2A12* most related pathways via single gene GSEA.

GO biological process (BP) functional enrichment analysis showed that the hub genes in the green module were mainly enriched in the transport of organic substance such as organic anions, and organic acids, including monocarboxylic and other carboxylic acids, across the blood–brain barrier and vessels. They could also play important roles in cardiac conduction regulation, establishment or maintenance of the transmembrane electrochemical gradient and potassium ion transmembrane transport (Fig. 4A). However, no statistically significant results were identified by KEGG analysis.

### Pathways associated with IA formation via GSVA

The pathway with logFC > 0.3 or logFC < -−0.3 was defined as an upregulated or downregulated pathway, respectively (Supplementary Table 3). Fifteen pathways related to immune processes were significantly activated in the IA group, whereas 7 metabolism pathways were inhibited according to the GSVA results (Fig. 4B).

### GSEA

According to normalized enrichment scores, genes in the IA group were mostly actively related to metabolism of propanoate and butanoate, “valine, leucine and isoleucine degradation”, “vascular smooth muscle contraction”, and “calcium signaling pathway”. Nevertheless, those genes were mostly negatively related to cell component pathways (“spliceosome”, “proteasome”, “lysosome”, etc.), immunity function (B and T-cell receptor signaling pathway, JAK/STAT signaling pathway, chemokine signaling pathway, etc.) (Fig. 4C).

### Hub genes validation and efficacy evaluation

**Figure 5:**
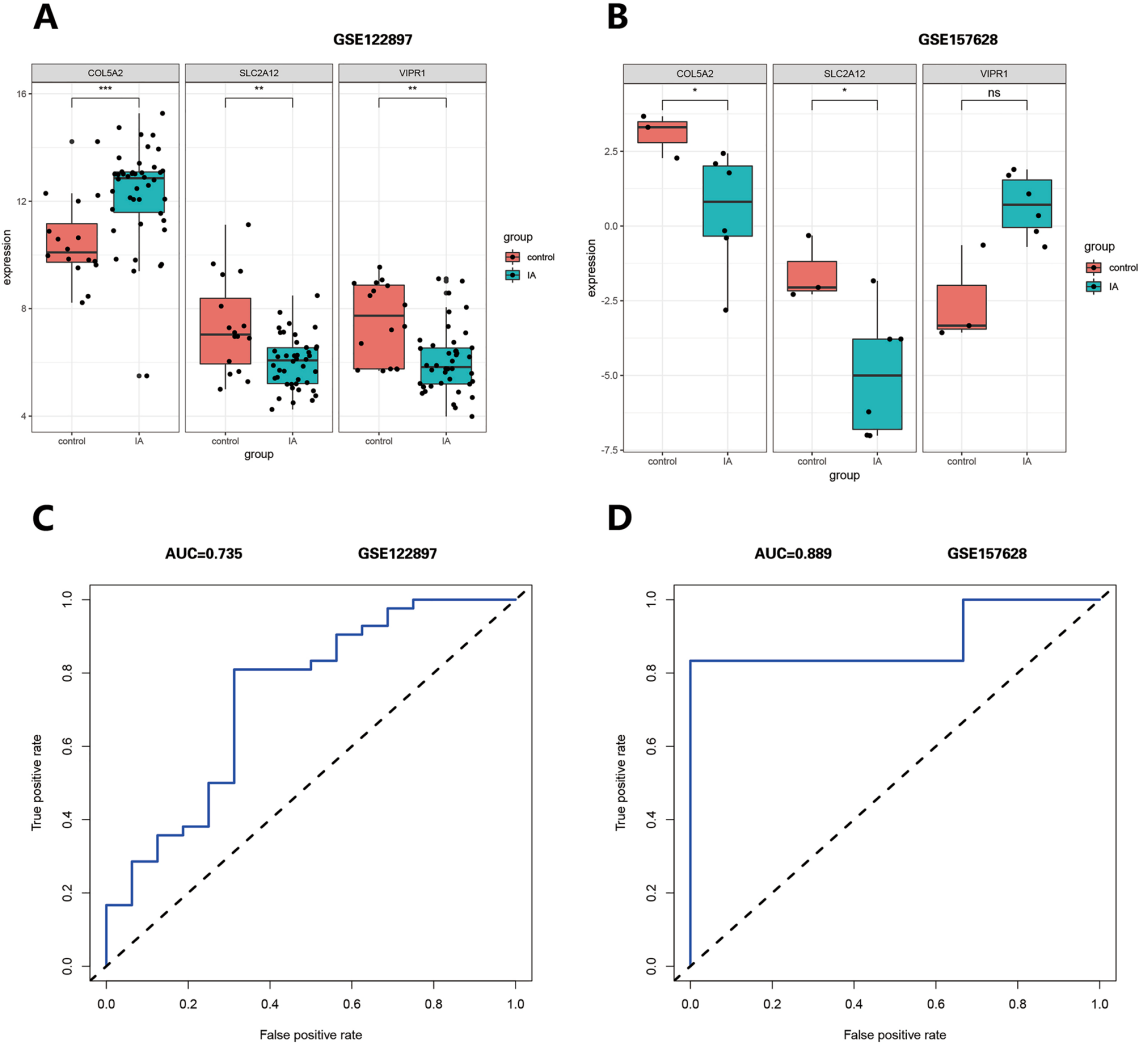
Expression of three hub genes in two datasets. (**A**) Expression boxplot of *COL5A2, SLC2A12* and *VIPR1* in GSE122897. (**B**) Expression boxplot of *COL5A2, SLC2A12* and *VIPR1* in GSE157628. **(C)** ROC plot of *SLC2A12* in GSE122897. (**D**) ROC plot of *SLC2A12* in GSE157628. (ns = no significance, *P < 0.05, **P < 0.01,***P < 0.001).

In the GSE122897 and GSE157628 datasets, the expression of *SLC2A12* was significantly decreased in IA patients (Fig. 5A, 5B). *COL5A2* and *VIPR1* were omitted as their expression patterns were different in those two datasets.

The ROC curve for the task of distinguishing IA samples from controls was plotted and the AUC was calculated. The AUCs of *SLC2A12* were greater than 0.7 in the two datasets (Fig. 5C, D). *SLC2A12* could differentiate IA from control in the two datasets.

### TFs, miRNAs and ferroptosis markers related to the key gene

Nine TFs were extracted from Enrichr (Supplementary Table 4), and 23 were extracted from hTFtarget (Supplementary Table 5). Overlap of them is *AR* and *NANOG*. The expression correlation analysis showed that *SLC2A12* was positively correlated with *AR* (Supplementary Image 2), whereas, there was no correlation with *NANOG* in GSE122897 (Supplementary Image 3).

There were 14 microRNAs targeting *SLC2A12,* including hsa-miR-335-5p, hsa-miR-574-5p, hsa-miR-1277-5p ,hsa-miR-190a-3p,hsa-miR-6867-5p,hsa-miR-223-5p,hsa-miR-767-5p,hsa-miR-502-3p, hsa-miR-501-3p,hsa-miR-5011-5p, hsa-miR-147a, hsa-miR-2113, hsa-miR-5010-3p, and hsa-miR-6818-5p, found in the GeneCard database. The mRNAs and miRNAs related to IA occurrence are shown in Supplementary Table 5. Among them, hsa-miR-223-5p and hsa-miR-502-3p overlapped with miRNAs targeting *SLC2A12* and were related to IA formation.

In the FerrDb database, we found that SLC2A12 was a deduced biomarker of ferroptosis which is a regulated form of cell death driven by dysfunction of the lipid repair enzyme glutathione peroxidase 4 (GPX4) and subsequent accumulation of lipid-based reactive oxygen species (ROS).

### External validation

**Figure 6:**
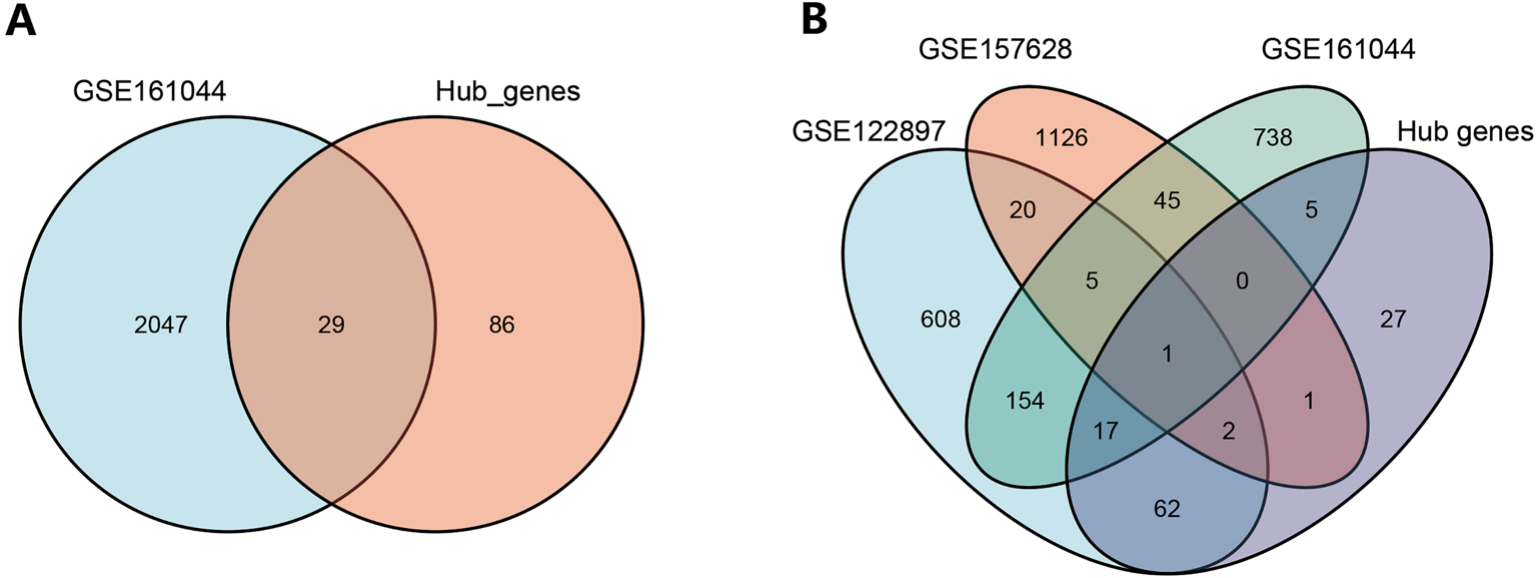
Venn diagrams (**A**) overlap of hub genes in green module and DEGs of GSE161044. (**B**) overlap of hub genes in green module and downregulated DEGs of three GEO datasets, GSE161044, GSE122897 and GSE157628. This figure was generated with the free application online, Venny 2.1 (https://bioinfogp.cnb.csic.es/tools/venny/index.html).

We found that 29 genes that were potential biomarkers for the occurrence of IA (Fig. 6A), overlapped with the hub genes of the green module and DEGs in GSE161044 (Supplementary Table 2). The common upregulated DEGs of GSE161044, GSE122897 and GSE157628 did not overlap with the hub genes (Supplementary Image 4). Only one hub gene, *SLC2A12,* overlapped with the downregulated DEGs of the three GEO datasets (Fig. 6B).

## Discussion

We considered two aspects when designing this study. On the one hand, in contrast to microarrays, RNA-seq technology has many unique advantages such as high sensitivity, directly detecting the sequence of each transcript fragment^10^. RNA-seq can be directly conducted on any species for transcriptional investigation without prior knowledge of its genetic information as it is not necessary to design a specific probe in advance. It provides multiple layers of resolution and transcriptome complexity, with less background noise and a broader dynamic range of RNA expression. RNA-seq is increasingly used in biotechnology and is expected to replace microarrays for detecting transcriptome profiles^11^. However, most publicly available RNA-seq data are in raw forms, not as friendly with researchers as microarray data for comprehensive analysis in silico.

On the other hand, global neuroscience studies tend to investigate the molecular mechanisms of IA formation by comparing cerebral IA samples with the STAs or MMAs. Gathering cerebral artery samples is challenging because there is almost no bypass in cerebral arteries except the Willis circle. Occlusion or resection of cerebral arteries will probably result in cerebral infarction. STA and MMA, which are derived from external carotid arteries, are replaceable and easy to acquire during neurosurgery. However, cerebrovascular diseases are rarely detected in the external carotid artery. Therefore, the pathogenesis and progression of IA should be studied, focusing on cerebral arteries per se. Therefore, we selected the two GEO datasets of *Homo sapiens*, GSE122897 and GSE157628 for the bioinformatic analysis of IA pathogenesis, and a GEO dataset of *Rattus norvegicus* for external validation.

Understanding the molecular functions of the hub genes in this study may contribute to IA diagnosis and treatment. We deduced 115 hub genes in the green module associated with IA onset. GO BP analysis showed that their functions were enriched in multiple organic substance transport. GSVA showed that 15 immune process pathways were activated and 7 metabolism pathways were inhibited in IA formation. This finding suggested that IA formation was involved in immunological processes accompanied by metabolic pathways. *SLC2A12* (also known as *GLUT12*), an insulin-independent glucose transporter, is the only ferroptosis-related gene among the hub genes in the green module; therefore, we selected it as a focus for our IA pathogenesis research.

To our knowledge, the loss and degeneration of vascular smooth muscle cells (VSMCs) are the major histopathological features of intracranial aneurysm^12^. VSMC death is controlled by multiple programmed cell death phenotypes, including ferroptosis^13^. For example, cigarette smoke extract can induce vascular smooth muscle cell death via ferroptosis^14^. IA onset is associated with VSMC death, which leads to thinning of the media and an increased rupture risk^15^. *SLC2A12* is expressed in VSMCs at both the mRNA and protein levels ^16^. Based on these facts, we discuss functions of *SLC2A12* and VSMC ferroptosis in IA pathogenesis below.

GLUT isoforms (GLUT1-12) comprised facilitative glucose transporters (GLUTs). Transcriptional control mechanisms for key GLUT isoforms in VSMCs have been reported^16^. SMC phenotypic switching plays a critical role in cerebral aneurysm formation, progression, and rupture^12^. The switch to the differentiated state manifested downregulation of *GLUT1, -9, and -10* mRNAs with an accompanying upregulation of *GLUT12* mRNA^16^. In addition, damaged brain vessels and reduced expression of glucose transporter 1 (GLUT1) in 5xFAD mouse brains were demonstrated by Kee-Chan Ahn^17^. Although the roles of GLUTs in the pathogenesis of intracranial aneurysms have not been elucidated clearly, we hypothesize that *SLC2A12* is related to IA formation.

Glucose metabolism dysfunction can induce ferroptosis in cells^18,19^. *SLC2A12* was deduced as a potential biomarker in the FerrDb database. The GeneCard database shows that SLC2A12 is a member of HIF1 alpha pathway, NRF2 pathway, mesodermal commitment pathway, and PAK pathway as well as pathways for the transport of glucose and other sugars, bile salts and organic acids, metal ions and amine compounds. Some of these pathways have been reported to be associated with ferroptosis in multiple tissues^20–22^. HIF1 alpha pathway plays an important role in neurocyte ferroptosis^20^. The NRF2 signaling pathway mediates lipid peroxidation and ferroptosis^21^. PAK surper pathway covers the antioxidant action of the vitamin C pathway, and vitamin C could significantly activate ferroptosis and inhibit anaplastic thyroid cancer cell growth^22^. In particular, the HIF1 alpha pathway has received sustained and extensive attention. It regulates the transcription of over 40 genes allowing adaptation to hypoxic conditions, by increasing oxygen delivery or facilitating metabolic adaptation to hypoxia. Proteins of the target genes include erythropoietin, glucose transporters, glycolytic enzymes, vascular endothelial growth factor, and other factors critical to vascularization, metabolic regulation, cell multiplication and survival. Its functions annotating how cells react to oxygen were made conspicuous by the 2019 Nobel Prize in Physiology or Medicine. These pathways can interact with those resulting from GSVA (Fig. 4B), such as the NOD-like receptor signaling pathway and P53 signaling pathway.

We applied single-gene GSEA with the guilt by association method to annotate the functions of *SLC2A12*. Single-gene GSEA was usually performed after samples were divided into two groups, the highly expressed group and the lowly expressed group, according to the median expression level of the selected gene. In this GSEA, a decreasingly ranked gene list based on correlation values with *SLC2A12* was used. Guilt by association approach in GSEA can deduce the enriched potential KEGG pathways *SLC2A12* involved in. We deduced that *SLC2A12* activated multiple organic substance metabolism pathways and suppressed immune response pathways including the JAK/STAT signaling pathway, chemokine signaling pathway, B and T-cell receptor signaling pathway. The roles of *SLC2A12* in metabolism and immune response pathways merit further research.

Misregulated ferroptosis can be implicated in multiple immune events^23^. Recently, Rui Kong reported that activation of the JAK/STAT pathway can induce ferroptosis in hepatocellular carcinoma cells^24^. In addition, the JAK/STAT pathway can participate in various biological processes in VSMCs^25^ and crosstalk with HIF1 alpha pathway^26,27^. To date, no evidence has interpreted the correlation between *SLC2A12* and the JAK/STAT signaling pathway. The functions of *SLC2A12* in immune activity should be explored in the future.

Based on the above literature review, *SLC2A12* could be a novel biomarker associated with the occurrence of IA. Its expression was significantly decreased in human and rat IA tissue as we have interpreted above. This would suppress organic substance transmembrane transportation and metabolism together with an enhanced immune response in cerebral arteries. Various immune molecular pathways are activated, especially JAK/STAT signaling and HIF1 alpha pathway.Via the VSMC ferroptosis phenotype, IA occurs and develops. Regulators of *SLC2A12* including TFs and miRNAs that we have found in this article, would supplement the understanding of the IA pathogenesis mechanism. External validation indicated that *SLC2A12* could be a candidate gene in further rat model experiments in order to confirm its biological functions in IA formation.

Limitations exist in this study. First, no experimental validation, using human tissue, was performed in our research due to obstacles in gathering human cerebral artery samples. Second, a small quantity of samples could cause variations in the results. Third, ruptured IAs and unruptured IAs have different gene expression profiles, which has been discussed in many studies. We synthesized and compared them with controls.

## Conclusions

In the present study we identified 115 hub genes related to the pathogenesis of IA onset and deduced their potential roles in various molecular pathways; this new information may contribute to IA diagnosis and treatment. By external validation, 29 of the hub genes are potential biomarkers associated with this process in both humans and rats. In particular, the *SLC2A12* gene may play important roles. The molecular involvement of SLC2A12 in IA pathogenesis can be further studied in a rat model.

## Supplementary Information

Supplementary Information 1.

Supplementary Information 2.

Supplementary Information 3.

Supplementary Information 4.

Supplementary Information 5.

Supplementary Information 6.

Supplementary Information 7.

Supplementary Information 8.

Supplementary Information 9.

Supplementary Information 10.

Supplementary Information 11.

Supplementary Information 12.

Supplementary Information 13.

Supplementary Information 14.

## Data Availability

The data used to support the current study are available from the corresponding author on reasonable request.

## Abbreviations

IA: Intracranial aneurysm
DEGs: Differentially expressed genes
RNA-seq: RNA sequencing
STA: Superficial temporal arteries
MMA: Middle meningeal arteries
GEO: Gene Expression Omnibus
GSVA: Gene-set variation analysis
GSEA: Gene set enrichment analysis
WGCNA: Weighted gene co-expression network analysis
ME: Module eigengene
MM: Module membership
GS: Gene significance
ROC: Receiver operating characteristic
AUC: Area under curve
GO: Gene ontology
KEGG: Kyoto encyclopedia of genes and genomes
GLUTs: Glucose transporters
TFs: Transcription factors
ceRNA: Competing endogenous RNA
