# Supplementary figures and images for: Bioinformatics analysis reveals potential biomarkers associated with the occurrence of intracranial aneurysms
Source: Sci Rep. 2022 Aug 2;12:13282. doi: 10.1038/s41598-022-17510-7 (PMC9345973; doi:10.1038/s41598-022-17510-7)

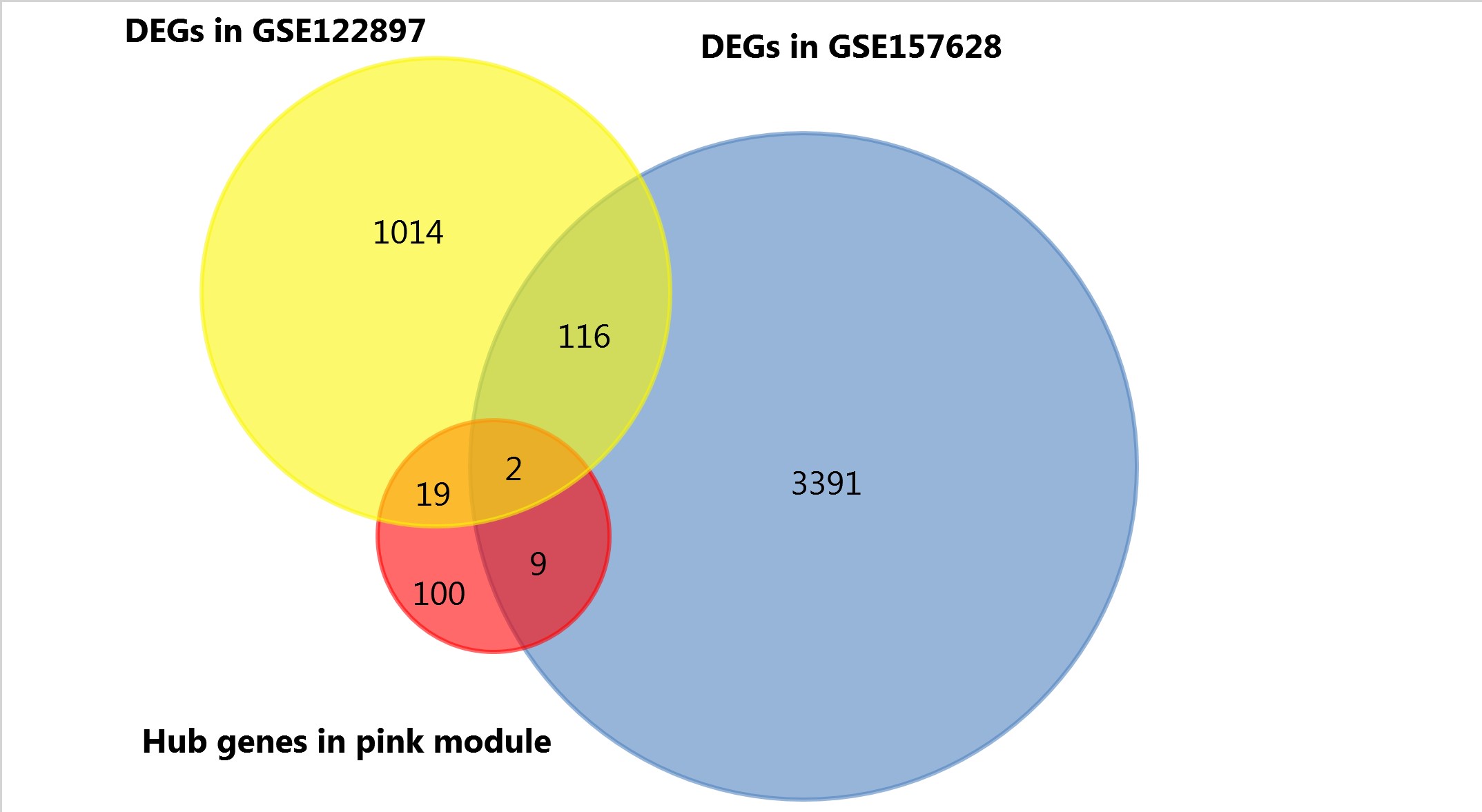

Supplement: Supplementary file 1 — Supplementary Information 1. [file 41598_2022_17510_MOESM1_ESM.jpeg]

GSE122897

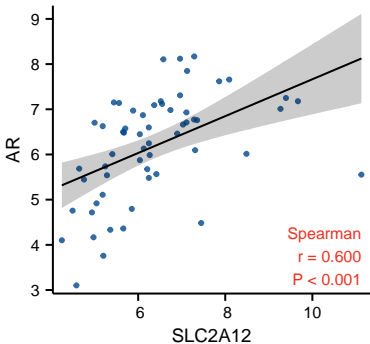

Supplement: Supplementary file 2 — Supplementary Information 2. [file 41598_2022_17510_MOESM2_ESM.pdf]

GSE122897

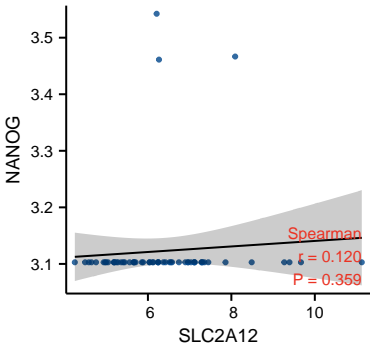

Supplement: Supplementary file 3 — Supplementary Information 3. [file 41598_2022_17510_MOESM3_ESM.pdf]

GSE157628

GSE161044

GSE122897

Hub genes

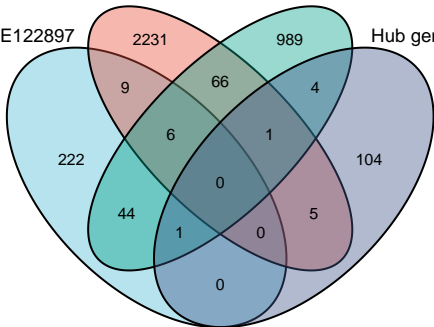

Supplement: Supplementary file 4 — Supplementary Information 4. [file 41598_2022_17510_MOESM4_ESM.pdf]
